# Pathological Society of Philadelphia

**Published:** 1842-10-15

**Authors:** 


### THE MEDICAL EXAMINER.

### PHILADELPHIA, OCTOBER 15, 1842.

### PATHOLOGICAL SOCIETY OF PHILADELPHIA.

At the annual election for officers, held September 24th, 1842, the following gentlemen were elected:

*President*, Nathaniel Chapman, M. D.

*Vice Presidents*, Jacob Randolph, M. D., Samuel George Morton, M. D., Charles D. Meigs, M. D.

*Secretary*, Thomas Stewardson, M. D.

*Treasurer*, George W. Norris, M. D.

*Curator*, Edward Hartshorne, M. D.

